# Do women in major cities experience better health? A comparison of chronic conditions and their risk factors between women living in major cities and other cities in Indonesia

**DOI:** 10.3402/gha.v8.28540

**Published:** 2015-12-16

**Authors:** Yodi Christiani, Julie E. Byles, Meredith Tavener, Paul Dugdale

**Affiliations:** 1 Research Centre for Generational Health and Ageing, University of Newcastle (Hunter Medical Research Institute), Newcastle, Australia; 2 Centre for Health Stewardship, Australian National University, Canberra, Australia

**Keywords:** urban health, women, chronic disease, BMI, hypertension, Indonesia

## Abstract

**Background:**

Inhabitants of rural areas can be tempted to migrate to urban areas for the type and range of facilities available. Although urban inhabitants may benefit from greater access to human and social services, living in a big city can also bring disadvantages to some residents due to changes in social and physical environments.

**Design:**

We analysed data from 4,208 women aged >15 years old participating in the fourth wave of the Indonesia Family Life Survey. Chronic condition risk factors – systolic and diastolic blood pressure (SBP and DBP), body mass index (BMI), and tobacco use – among women in four major cities in Indonesia (Jakarta, Surabaya, Medan, and Bandung) were compared against other cities. Fractional polynomial regression models were applied to examine the association between living in the major cities and SBP, DBP, BMI, and tobacco use. The models were also adjusted for age, education, employment status, migration status, ethnic groups, and religion. The patterns of SBP, DBP, and BMI were plotted and contrasted between groups of cities.

**Results:**

Chronic condition prevalence was higher for women in major cities than in contrasting cities (*p*<0.005). Living in major cities increased the risk of having higher SBP, DBP, BMI and being a current smoker. Chronic disease risk factors in major cities were evident from younger ages.

**Conclusions:**

Women residing in Indonesia's major cities have a higher risk of developing chronic conditions, starting at younger ages. The findings highlight the challenges inherent in providing long-term healthcare with its associated cost within major Indonesian cities and the importance of chronic disease prevention programmes targeting women at an early age.

Urbanisation creates opportunities for people to gain better access to employment and to human and social services. Whereas two decades ago fewer than 40% of the world's population lived in cities, today this figure is greater than 50% (1). The proportion is expected to continue to increase, so that by 2050 the proportion of people living in urban areas will reach 70% of the total global population (2).

UN-Habitat recorded that, globally, more than half the people who moved from rural to urban areas during 2008–2009 were women (3). Women are attracted to living in major cities, believing that moving may improve their status and position and provide opportunities for their children to receive a better education and gain employment (4). However, most of these women end up living in urban areas where there are dangers concerning their safety or where access to resources and services are limited (4), which could lead to poor health outcomes.

Studies show that urban populations have better health on average than non-urban populations (5, 6). However, health inequalities exist between cities in a particular country (7–9), because different urban conditions could result in different health status. Urban living conditions can lead to poor health outcomes. For example, poor air quality in urban areas could lead to an increasing rate of lung diseases, and worsening water and waste management could lead to a rise in infectious diseases (10). Urban areas are also usually more socially heterogeneous than rural areas and are composed of more racial/ethnic groups, which could lead to social segregation and social strain. These factors make cities more vulnerable to social conflict, violence, and crime (11). Furthermore, people migrating from rural areas to big cities also face a range of social problems that impact on health – examples of which include assimilation to their place of settlement, new and different job conditions, and barriers to healthcare access (12).

Today, chronic disease has replaced infectious disease as the main specific cause of death among women as a result of their physical and social environments (13). Many cities, particularly in low income countries, were developed without due attention to long-term planning for their inhabitants (14), resulting in a lack of open spaces for physical activity. Consequently, residing in urban environment increases women's odds of being overweight (7, 15, 16). The social environment and lifestyle in big cities may place women at a higher risk of engaging in unhealthy behaviours, including excessive alcohol consumption, smoking, and unhealthy diet. Combined with a sedentary lifestyle, such behaviours can lead to a higher prevalence of obesity and hypertension (15, 17, 18), which are major risk factors for chronic diseases such as cancer, cardiovascular disease, and cerebrovascular disease.

## Urban women's health in Indonesia

In the last few decades, Indonesia has achieved significant improvement in overall health status. One of the indicators of this improvement is life expectancy. Over the last three decades, life expectancy has increased from 46 years (1971) to 70.5 years (2007) (19–21). This improvement has altered the population pyramid in Indonesia, with increasing numbers of older people. At the same time, chronic disease morbidity has also increased, with people in older age groups being the most vulnerable to chronic disease. Currently, Indonesia is facing a double burden of disease, where non-communicable disease is starting to emerge while communicable disease is still prevalent (22). Estimates indicate that since 2002 cardiovascular disease has been the main cause of death in Indonesia (23). In 2007, a national survey conducted by the Ministry of Health of Indonesia found a high prevalence of chronic conditions, with hypertension being the most prevalent (32%) (19).

Consistent with many other developing countries, the proportion of people living in urban areas in Indonesia has gradually increased – from 42% of the population in 2001 to 51% in 2011 – with most people residing in the major cities (24). The population density in Jakarta reached 14,695/km^2^ in 2010 (25). Big cities offer access to economic opportunities and human and social services. People believe that moving to a major city such as Jakarta will improve their status, position, and opportunities (4). However, in major cities, the complex dynamic of multicultural groups, differences in socio-economic structure, and educational attainment has brought a bigger challenge to urban healthcare provision (26). In Indonesia, local governments have the authority to develop policy at the local level, which can influence the health status of the cities’ inhabitants and can also lead to health inequalities between different cities across the country.

Few studies have assessed the health of Indonesia's urban-dwelling women. However, the prevalence of various chronic conditions, such as asthma, cancer, diabetes, hypertension, and stroke, are greater in urban compared to rural areas and higher among women than men (19). To further explore urban women's chronic conditions in Indonesia, we conducted the current study to examine differences between different cities and the prevalence of chronic conditions and their major risk factors – blood pressure, body mass index (BMI), and current smoking status among urban women in Indonesia.

## Methodology

### Population data

We conducted secondary analysis of data from the fourth wave of the Indonesia Family Life Survey (IFLS), conducted in 2007/2008. The IFLS was first conducted in 1993 and has been repeated three times, in 1997/1998, 2000, and 2007/2008 (IFLS-4) (27). The sampling and survey strategies utilised in the IFLS have been described in detail elsewhere (27, 28). Briefly, the IFLS assesses multiple indicators at both the individual and household levels. It covers the 13 most populated provinces out of 27 provinces in Indonesia, which account for around 83% of the population based on 1991 population statistics (27). Over the time, there have been changes in the government administrative structures in Indonesia affecting a number of provinces in Indonesia. Although the survey covers the same geographical areas, the IFLS-4 was conducted in 20 of the 33 provinces in Indonesia. They are seven provinces in Sumatra Island (North Sumatra, West Sumatra, Riau, South Sumatra, Lampung, Bangka Belitung, and the Riau Islands), six provinces on Java Island (DKI Jakarta, West Java, Central Java, Yogyakarta, East Java, and Banten), three provinces in Kalimantan (Borneo) Island (Central Kalimantan, South Kalimantan, and East Kalimantan), two provinces in Sulawesi (North Sulawesi and South Sulawesi), Bali, and West Nusa Tenggara.

The IFLS-4 data are open for public use with prior registration on the study website (www.rand.org/labor/FLS/IFLS/ifls4.html). Approval for the IFLS-4 project was granted by the institutional review boards at RAND and Gadjah Mada University, Yogyakarta, Indonesia. The use of the data set for this study was approved by the Human Research Ethics Committee, University of Newcastle, Australia.

### Measures

#### Chronic conditions and risk factors

Chronic conditions were ascertained in the IFLS-4 by asking whether participants had ever been diagnosed with any of the following conditions: physical disabilities, brain damage, vision problems, hearing problems, heart problems, or depression. In this study, those who had ever been diagnosed with any of these conditions were coded as having a chronic condition. Further, for participants aged 40 years or over, the IFLS-4 assessed whether the participants had ever been diagnosed with hypertension, diabetes, asthma, lung disease, liver disease, stroke, or cancer.

We used the average of three blood pressure measurements as the values for systolic blood pressure (SBP) and diastolic blood pressure (DBP). BMI was calculated from weight and height measurements conducted at the same time as the blood pressure measurement. Current smoking status was based on participants’ response to questions related to tobacco use.

#### Main predictors

*Groups of cities*. In this study, cities where the IFLS-4 was conducted were classified into two groups: major cities and contrasting cities. By this classification a major city is 1) the national or provincial capital city; 2) surrounded by satellite cities, representing its status as a centre of business and economic activities; and 3) heterogeneous in population. Following these criteria, we classified Jakarta, Surabaya, Medan, and Bandung as major cities. These are the four largest cities in Indonesia, with each described briefly below.

1. Jakarta is the capital city of Indonesia and is the most populous city in Indonesia. As the capital city of Indonesia, DKI Jakarta has become the centre of national government, the economy, development, and education in the education in the country. Main economic sectors include services and trading, hotels and restaurants, and manufacturing (29).
2. Surabaya, the capital city of East Java Province, is located on the northern coast of East Java. The inhabitants of Surabaya come from different ethnic groups in the country. They are Malay, Chinese, Arabic, Sundanese, Batak, Dayak, and Balinese, and the majority ethnic groups are native Surabaya and Maduranese (30). With vast growth in trade and economic development, the city's economic growth is greater than both the provincial and national economic growth (30).
3. Medan is the third largest city in Indonesia, after Jakarta and Surabaya. Of the four largest cities in the country, Medan is the only city located outside of Java Island. As the capital city of North Sumatra Province, Medan is the centre of provincial government administration, the economy, communication, tourism, and regional trading. Currently there are 86 national companies and 17 international companies operating in industrial locations in the city (31).
4. Bandung is the capital city of West Java Province. Located not far from Jakarta, Bandung is a centre of education, industry, and tourism. It is where 437 tertiary education institutions, 24 shopping malls, and more than 9,000 stores are located (32). The city provides not only opportunity for big industries but also for the informal industrial sector, such as the production of leather, clothes, arts, trading goods, pulp, and paper. These opportunities have attracted people from other areas of the country to migrate to Bandung, and consequently Bandung has become a multi-ethnic city (32).

The other cities in 20 provinces surveyed in the IFLS-4, which did not meet the criteria for major cities, were classified as contrasting cities. Cities included in this group were less populated and included cities that cover suburban or rural areas, cities surrounded by rural areas, or district capital cities. They include newly developed cities, cities with wider suburban areas compared to the major cities, and those with more homogenous backgrounds than the major cities.

#### Other predictors

We included age, educational attainment (primary, secondary, or tertiary), five quintiles of household wealth based on household assets, employment status (being in paid work vs not in paid work), in-country migration (migrant vs non-migrant), ethnic background (Javanese vs non-Javanese), and religion (Moslem vs non-Moslem) as predictor variables.

### Method

We compared the prevalence of chronic conditions and current smoking among women in major cities and contrasting cities. Weighted prevalence was calculated using the sampling weight factor constructed by RAND (27) to allow use of the dataset for cross-sectional analysis. Independent *t*-tests were applied to contrast the mean of SBP, DBP, and BMI. We then applied fractional polynomial (FP) regression models to examine the association between living in major cities and hypertension, obesity, and tobacco use, adjusted with other predictors described above. Next, we plotted SBP, DBP, and BMI against the predicted line across age in both settings to further contrast the pattern of chronic disease risk factors across ages in major and contrasting cities.

The FP model was introduced by Royston and Altman in 1994 (33, 34). It measures the association between predictors and outcome variables at a detailed level and acknowledges that categorisation of continuous data may produce bias, particularly where relationships are non-linear with different cut-off points producing different associations (35).

In FPs, Royston and Altman propose a fixed generalisation of the power *p*, which is chosen from the set {−2, −1, −0.5, 0, 0.5, 1, 2, 3} (33). This generalisation defines the first-degree FP equation with power *p*_*1*_ as FP1(*p*_1_)=*α*+β_1_X^*p*1^ and the second-degree FP equation with power *p*_*1*_ and *p*_*2*_ as FP2(*p*_*1*_,*p*_*2*_)=*α*+β_1_X^*p*1^+β_2_X^*p*2^. Hence, the general equation of FP is denoted as follows:

$$y=a+\sum_{j=1}^{m}p_{j}x^{pj}$$

where *m*=3, and *p* is chosen from the set {−2, −1, −0.5, 0, 0.5, 1, 2, 3} (34).

## Results

### Prevalence of chronic conditions and risk factors

A total of 4,208 women aged 15 years and above were included in the analysis. Of these women, 1,400 were residing in the major cities, and the other 2,808 were residing in the contrasting cities. The mean age was 36.1 years old (SD=15.1). The mean age in major cities was significantly younger than in contrasting cities (35.1 years old vs 36.6 years old, *p*<0.05), although the difference was not large.

Table 1 shows the comparison of chronic conditions and risk factors among women in major cities and contrasting cities. The prevalence of chronic conditions among women in major cities was significantly higher than those in contrasting cities (17.5% in major cities, 12.0% in contrasting cities, *p*<0.001), with the prevalence of current smoking almost doubled in the major cities (*p*<0.05).

**Table 1:** Chronic conditions and risk factors among women, by group of cities

	Prevalence or mean (SD)				
			
Outcome variable	Major (*N*=1,400)	Contrasting (*N*=2,808)	Total (*N*=4,208)	*X* ^2^ or *t*	*p*
Has a chronic condition (%)	17.5%	12.0%	13.7%	19.241	<0.001
SBP in mmHg [mean (SD)]	125.0 (19.7)	124.5 (19.9)	124.7 (19.8)	−0.667	0.504
DBP in mmHg [mean (SD)]	80.1 (9.4)	78.9 (9.8)	79.5 (9.7)	−5.274	<0.001
BMI in kg/m^2^ [mean (SD)]	24.0 (4.5)	23.7 (4.4)	23.8 (4.4)	−2.232	0.026
Current smoker (%)	4.3%	2.4%	3.0%	9.249	0.002

SBP, systolic blood pressure; DBP, diastolic blood pressure; BMI, body mass index.

As shown in Table 2, the prevalence of chronic disease among women aged 40 years and older in major cities was higher than those in the contrasting cities (51.9% in major cities, 40.8% in contrasting cities; *p*<0.001). In general, hypertension, uric acid/gout, and arthritis/rheumatism were the three most prevalent diagnoses among women aged 40 years old in the cities. The prevalence of hypertension, diabetes, lung conditions other than asthma, and uric acid were significantly higher in the major cities than in the contrasting cities (*p*<0.05).

**Table 2:** Chronic conditions among women aged 40 years and above, by group of cities

	Prevalence (%)				
			
Chronic disease	Major (*N*=467)	Contrasting (*N*=1,011)	Total (*N*=1,478)	Chi-square	*p*
Hypertension	35.1	23.0	26.5	21.913	<0.001
Diabetes	8.7	4.1	5.5	11.354	<0.001
Asthma	3.7	2.7	3.0	1.054	0.306
Other lung conditions	2.7	1.0	1.5	5.718	0.017
Heart problems	3.3	3.7	3.6	0.161	0.688
Liver disease	1.3	0.9	1.0	0.325	0.569
Stroke	1.7	1.1	1.3	0.922	0.337
Cancer	1.9	0.7	1.0	3.288	0.070
Arthritis/rheumatism	12.6	11.9	12.1	0.136	0.712
Uric acid/gout	14.6	9.2	10.7	8.696	0.003
Any of the conditions above	51.9	40.8	44.0	14.477	<0.001

### Factors associated with SBP, DBP, BMI and being a current smoker

The association between living in a major city and chronic disease risk factors are shown in Table 3. The models were adjusted for other predictors. Age was kept as a continuous variable in the FP models (Table 4).

**Table 3:** Predictors of SBP, DBP, BMI, and smoking status among women residing in the cities

	SBP		DBP		BMI		Current smoker	
				
Predictors	Coef	95% CI	Coef	95% CI	Coef	95% CI	Coef	95% CI
Major cities (reference: contrasting cities)	1.325	(0.263 to 2.386)	1.718	(1.107 to 2.329)	0.340	(0.067 to 0.614)	0.796	(0.428 to 1.163)
Age								
Age_1^a^	0.670	(0.609 to 0.732)	1.732	(1.500 to 1.964)	11.459	(10.335 to 12.583)	−12.695	(−16.828 to −8.563)
Age_2^a^	−0.281	(−0.311 to −0.251)	−0.759	(−0.871 to −0.646)	−0.253	(−0.287 to −0.220)		
Education (reference: primary or less)								
Secondary	−2.292	(−3.616 to −0.968)	−0.439	(−1.203 to 0.325)	−0.362	(−0.702 to −0.021)	0.359	(−0.067 to 0.786)
Tertiary	−2.364	(−4.108 to −0.620)	0.406	(−0.601 to 1.413)	−0.313	(−0.760 to 0.135)	−0.394	(−1.187 to 0.399)
Household wealth (reference: Quintile 1)								
Quintile 2	−0.453	(−2.023 to 1.117)	−0.369	(−1.271 to 0.533)	0.519	(0.116 to 0.922)	−0.609	(−1.156 to −0.063)
Quintile 3	−1.161	(−4.108 to −0.620)	−0.390	(−1.288 to 0.508)	0.511	(0.109 to 0.913)	−0.833	(−1.408 to −0.258)
Quintile 4	−1.339	(−2.936 to 0.399)	−1.088	(−2.010 to −0.166)	0.696	(0.286 to 1.106)	−0.599	(−1.158 to −0.040)
Quintile 5	−0.780	(−2.412 to 0.257)	−1.275	(−2.216 to −0.334)	0.819	(0.399 to 1.238)	−0.632	(−1.204 to −0.059)
Being in paid work (reference: not in paid work)	−1.950	(−2.961 to 0.851)	−1.067	(−1.654 to −0.481)	−0.275	(−0.538 to −0.013)	0.204	(−0.164 to 0.571)
Migrant (reference: non-migrant)	−0.542	(−1.638 to 0.553)	−0.659	(−1.290 to −0.028)	−0.156	(−0.437 to 0.125)	0.113	(−0.319 to 0.545)
Non-Javanese background (reference: Javanese background)	0.053	(−0.969 to 1.074)	−0.133	(−0.721 to 0.456)	−0.070	(−0.333 to 0.193)	0.655	(0.247 to 1.064)
Moslem (reference: non-Moslem)	2.277	(0.685 to 3.869)	1.891	(0.971 to 2.810)	0.089	(−0.324 to 0.501)	0.322	(−0.322 to 0.966)

a Age is transformed in fractional polynomial model (see Table 4). SBP, systolic blood pressure; DBP, diastolic blood pressure; BMI, body mass index; CI, confidence interval, Coef=Coeficient.

**Table 4:** Fractional polynomial analysis of the effect of age on SBP, DBP, BMI, and smoking status among women residing in the cities, adjusted for education, household wealth, employment status, migration status, ethnic background, religion, and cities

	SBP	DBP	BMI	Current smoker
Degree of freedom (df)	4	4	4	2
Power	3 3	2 2	0.5 2	**−**2
Transformed covariate (age)				
Age_1	*X* ^3^−46.225	*X* ^2^−13.009	*X* ^0.5^−1.895	*X* ^−2^−0.076
Age_2	*X* ^3^*ln(X)−59.067	*X* ^2^*ln(X)−16.688	*X* ^2^−12.901	

*X*=age/10. SBP, systolic blood pressure; DBP, diastolic blood pressure; BMI, body mass index; CI, confidence interval.

As shown in Table 3, after adjustment for other predictors, living in major cities increased the risk of having higher SBP, DBP, and BMI. Other predictors, such as age, having less education, and religion were also shown to have significant association with SBP and DBP. In respect to household wealth, only those grouped in the third quintile had significantly reduced SBP when compared to those grouped in the first quintile. In addition, having higher economic status also had a positive association with higher BMI, while women who were in paid work had smaller odds of having higher BMI.

Living in major cities also increased the odds of being a current smoker. Other predictors associated positively with being a current smoker included being a younger woman, being in the lower quintiles of household wealth, and having non-Javanese background.

### The predicted SBP, DBP, and BMI in major cities and contrasting cities

**Fig. 1:**
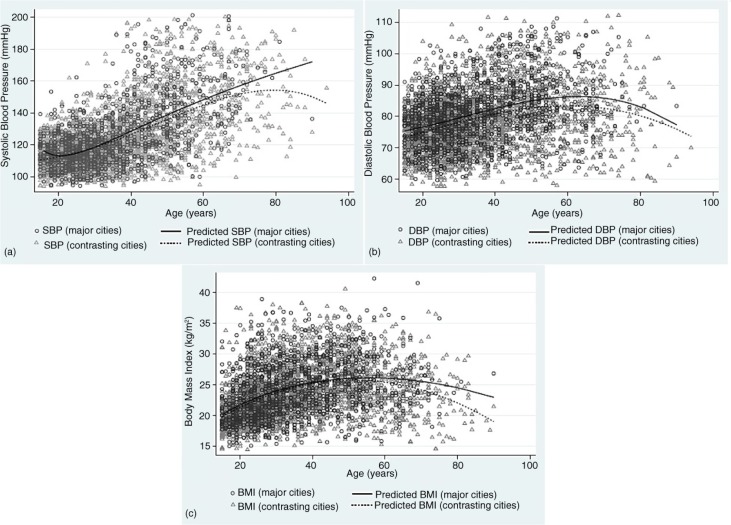
Distribution plot and predicted line of (a) systolic blood pressure, (b) diastolic blood pressure, and (c) body mass index among women ≥15 years old, by group of cities.

The predicted SBP, DBP and BMI for women in major cities were higher across age groups than those living in contrasting cities (Fig. 1). Pre-hypertension (SBP between 120 and 130 mmHg or DBP between 80 and 90 mmHg) started to emerge among women 30 years of age in major cities and around 38 years old in contrasting cities (Fig. 1a and b). The gaps in predicted SBP and BMI between major cities and contrasting cities were also wider across ages (Fig. 1a and c).

## Discussion

This study found that the prevalence of chronic conditions among women in major cities was significantly higher than those in contrasting cities (17.5% vs 12%), even though the mean age in the major cities was younger than in contrasting cities (35.1 years old vs 36.6 years old). Among women age 40 years and above living in the cities, the prevalence of chronic disease in major cities was significantly higher than in contrasting cities. Hypertension was indicated as the most prevalent chronic condition, followed by degenerative joint disease, such as gout and arthritis, and lifestyle-related chronic disease, such as diabetes and heart problems. In Indonesia, hypertension is the most frequently diagnosed condition by health centre among the elderly population, and urban life has the potential to increase the risk of having high blood pressure (36). This finding might be related to different physical and social conditions in major cities, compared to contrasting cities, that lead to an increasing risk of developing chronic conditions (11). Another possibility is that the higher prevalence of disease in major cities could also be a result of better health access in these cities compared to the contrasting cities and a greater chance of diagnoses being recorded and reported in the major cities. Attempting to examine this issue, this study examined the association between living in major cities and objective measurement of chronic disease risk factors, such as SBP, DBP, and BMI. Current smoking status was also assessed, acknowledging tobacco use as one of the major risk factors for chronic disease.

After adjustment for other predictors, the study found that women who were residing in major cities had a higher probability of high blood pressure (SBP and DBP), higher BMI – which in turn resulted in a higher probability of being overweight – and being a current smoker. Knowing that these three conditions are major risk factors for chronic disease, there is a greater chance that women in major cities have a higher probability of having chronic disease as well. Access to healthy food, rapid shifts in income, and changes in occupation types among the inhabitants of big cities could lead to a shift in lifestyle towards less physical activity and unhealthy dietary habits (37, 38).

In addition, as suggested by Levine (14), the high prevalence of chronic conditions among urban women is partly a result of physical and social changes in urban areas. Lack of open spaces and poor transportation systems reduce opportunities for urban women to engage in physical activity, thereby increasing their risk for developing obesity, which in turn leads to increased risk of chronic conditions (14). In the developing world, there is a lack of long-term planning in most cities. For example, in order to facilitate the increasing number of automotive vehicles, the transportation policy has been undermining the importance of bicycle lanes and sidewalks for pedestrians (39). Major cities also have a greater chance of having wider residential segregation, which leads to inequality of access to do physical activities for people living in certain environments or parts of the cities (40). Residential location could also determine access to healthcare and hence health conditions (8, 41).

With the higher odds of having hypertension, becoming overweight, and being a current smoker for women in the major cities compared to the contrasting cities, there is an indication of an increasing burden of chronic disease in the major cities over time. Hence, the difference in chronic disease prevalence between major and contrasting cities will be widened further. These findings highlight the importance of chronic disease risk factor screening at an early stage. It is particularly important given that chronic disease has now become the highest burden of disease among urban residents, with particular relevance to urban women.

Compared to contrasting cities, the ages of women having pre-hypertension in major cities – SBP between 120 and 130 mmHg or DBP between 80 and 90 mmHg (42) – were found to be younger. In addition, the predicted BMI across ages was also slightly higher for women in major cities from an early age. We argue that the social environment in big cities influences the behaviour of young people, which puts them at a higher risk of having chronic disease at an early age. The behavioural patterns among young people in the cities could be altered due to the availability of fast food and advanced technology, leading to inactive behaviour (37, 43).

Socio-economic status, reflected by educational attainment and household wealth, was another factor found to be a significant predictor for high blood pressure, having higher BMI, and being a current smoker. Women in the lower economic group had a higher risk of having hypertension and becoming a smoker. On the contrary, women with higher economic status had a higher risk of being overweight – reflected by higher BMI. It was also shown that well-educated women had a lower risk of being overweight. These findings supported another cross-sectional study among urban dwellers in China, where women with a lower level of education and higher level of income had a higher risk of being obese (7). With reference to health promotion, the findings indicate the importance of developing suitable health promotion materials and methods that are accessible by all groups of urban women, despite their socio-economic class. In addition, our findings also supported the importance of health promotion planners developing their campaigns to be relevant to their city.

This study involves a cross-sectional analysis that provides comparisons between women in different groups of cities, but with limitations in describing the exact patterns of urban living (including the period of living in the cities) and the development of disease. Despite its limitations, the study provides evidence on the emergence of non-communicable disease among women in Indonesia's cities, emphasising the importance of providing a women's health programme that extends beyond reproductive health.

## Conclusions

This study has shown that even though major cities may offer more access to social and human services compared to other settings, women residing in Indonesia's major cities do not have better chronic condition health outcomes than those who live in the contrasting cities. Better access to healthcare among women in major cities could provide opportunities for better Non-communicable Disease (NCD) control, with a gender-responsive NCD control programme at the primary level. Additionally, in order to expand the NCD control programme among women, integration of the NCD programme with other established primary healthcare programmes, such as family planning and maternal health, is worth considering.

With a higher probability for having hypertension and obesity and being a current smoker in the major cities – starting from an early age – we can expect a higher burden of chronic conditions among this population group, into the future. Our findings highlight the challenge of providing long-term healthcare, with its associated financial cost, in the major cities of Indonesia. The ageing population and the importance of a robust chronic disease prevention programme will make this an imperative. Therefore, in an era of decentralised health systems, it will be necessary for local governments to emphasise both healthcare provision and health promotion – targeting women at an early age – to prevent non-sustainable health costs into the future and incorporating ageing health into the current public health agenda.
